# Correction: Metformin-repressed miR-381-YAP-snail axis activity disrupts NSCLC growth and metastasis

**DOI:** 10.1186/s13046-022-02545-5

**Published:** 2022-12-09

**Authors:** Dan Jin, Jiwei Guo, Yan Wu, Weiwei Chen, Jing Du, Lijuan Yang, Xiaohong Wang, Kaikai Gong, Juanjuan Dai, Shuang Miao, Xuelin Li, Guoming Su

**Affiliations:** 1 Clinical Medicine Laboratory, Binzhou Medical University Hospital, Binzhou, 256603 People’s Republic of China; 2 Cancer research institute, Binzhou Medical University Hospital, Binzhou, 256603 People’s Republic of China; 3 Department of Thyroid and Breast Surgery, Binzhou Medical University Hospital, Binzhou, 256603 People’s Republic of China; 4 Department of Nursing, Binzhou Polytechnic University, Binzhou, 256603 People’s Republic of China

**Correction: *J Exp Clin Cancer Res *39, 6 (2020)**

**https://doi.org/10.1186/s13046-019-1503-6**

Following publication of the original article [1], the authors identified an error in Fig. 2, as the quality for the WB bands showed in the published paper are low, leading to some confusion about the results we provided the uncropped original data for the Fig. 2c and d.

**Fig. 2:**
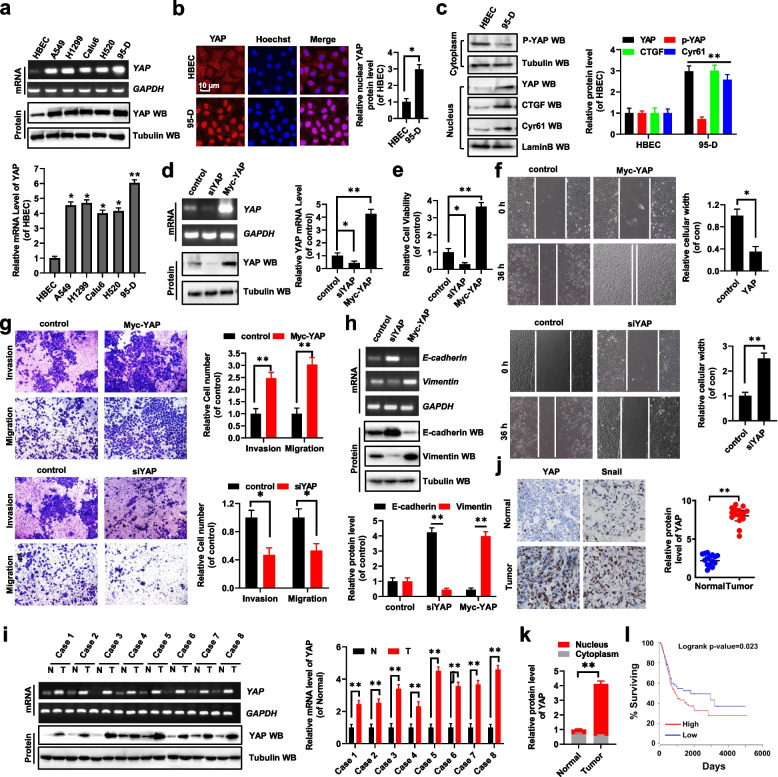
Higher expression of YAP promotes cellular growth, migration, invasion and EMT. **a** The expression of YAP was higher in NSCLC cell lines compared with their control cell line, HBEC, analyzed by RT-PCR, western blot and qRT-PCR assays. **b** The protein level of nuclear YAP was higher in high metastasis lung cancer cell line 95-D than its control cell HBEC by the immunofluorescent staining assay. **c** Immunoblotting with densitometric quantitation demonstrating increased nuclear Yap and decreased p-YAP in 95-D cells than its normal cell line meanwhile YAP’s target genes, CTGF and Cyr61, were higher in 95-D cells. **d-h** A549 cells were transfected with siYAP or Myc-YAP, respectively. **d** The expression of YAP was analyzed by RT-PCR, Western blot and qPCR assays. **e** The cellular viability was analyzed by CCK8 assay. **f** Cellular migration growth was analyzed by scratch assay. **g** The cellular invasion growth was analyzed by transwell assay. **h** The expressions of E-cadherin and Vimentin were analyzed by RT-PCR, western blot assays. **i** The expression of YAP was higher in human lung cancer tissues compared with their normal adjacent lung tissues analyzed by RT-PCR, western blot and qRT-PCR assays (*n* = 8). **j** Immunohistochemical (IHC) assay of the expression of YAP and Snail in the human lung cancer tissues and their normal adjacent lung tissues (*n* = 15). **k** YAP was higher in nucleus from lung tumor tissues than their normal adjacent lung tissues (*n* = 15). **l** Kaplan Meier overall survival (OS) curves of YAP (*p* = 0.023 by log-rank test for significance) for human lung cancers. Results were presented as mean ± SD, and the error bars represent the SD of three independent experiments. *P < 0.05 or **P < 0.01 indicates a significant difference between the indicated groups (two-tailed, unpaired Student’s t-test or one-way analysis of variance)

When the images were exported, they were compressed leading to the similar and repeated features within the bands. Moreover, to remove the confusion, we provided the uncropped original data for the Fig. 2c and d.

This correction does not change the result, interpretation, and conclusions of the study.
